# Association of serum 25-hydroxyvitamin D levels with all-cause and cause-specific mortality among postmenopausal females: results from NHANES

**DOI:** 10.1186/s12967-023-04413-y

**Published:** 2023-09-16

**Authors:** Jia-Wei Shi, Jiang-Nan Wu, Xiao-Yong Zhu, Wen-Hui Zhou, Jin-Ying Yang, Ming-Qing Li

**Affiliations:** 1 Shanghai Key Laboratory of Female Reproductive Endocrine Related Diseases, Hospital of Obstetrics and Gynecology, Fudan University, Shanghai, 200080 People’s Republic of China; 2 Longgang District Maternity &, Child Healthcare Hospital of Shenzhen City, Longgang Maternity and Child Institute of Shantou University Medical College, Shenzhen, 518172 People’s Republic of China; 3 Clinical Epidemiology, Hospital of Obstetrics and Gynecology, Fudan University, Shanghai, 200011 People’s Republic of China; 4 Medical Center for Human Reproduction, Beijing Chao-Yang Hospital, Capital Medical University, Beijing, 100043 People’s Republic of China; 5 Longgang Maternity and Child Clinical Institute of Shantou University Medical College, Shenzhen, Guangdong 518172 People’s Republic of China; 6 Laboratory for Reproductive Immunology, Hospital of Obstetrics and Gynecology, Fudan University, Shanghai, 200080 People’s Republic of China

**Keywords:** 25-hydroxyvitamin D, Postmenopausal female, Mortality, Cardiovascular disease, Cancer

## Abstract

**Background:**

Vitamin D deficiency is common among the population, but its relationship with mortality of postmenopausal females is unclear. The aim of this study is to explore the association between serum 25-Hydroxyvitamin D (25(OH)D) and all-cause and cause-specific mortality among postmenopausal women in the United States.

**Methods:**

6812 participants of postmenopausal females from the National Health and Nutrition Examination Survey (2001–2018) were included in this study. The mortality status of the follow-up was ascertained by linkage to National Death Index (NDI) records through 31 December 2019. We used cox proportional hazards models to estimate the association of serum 25(OH)D concentrations and mortality of postmenopausal females.

**Results:**

The mean level of serum 25(OH)D was 72.57 ± 29.93 nmol/L, and 65.34% had insufficient vitamin D. In postmenopausal females, low serum 25(OH)D concentrations were significantly associated with higher levels of glycohemoglobin, glucose, and lower levels of HDL. During follow-up, 1448 all-cause deaths occurred, including 393 cardiovascular disease (CVD)-related deaths and 263 cancer deaths. After multivariate adjustment, higher serum 25(OH)D levels were significantly related with lower all-cause and CVD mortality. In addition, serum 25(OH)D presented a L-shaped relationship with all-cause mortality, while appeared a U-shaped with CVD mortality, and the cut-off value is 73.89 nmol/L and 46.75 nmol/L respectively.

**Conclusions:**

Low serum 25(OH)D levels are associated with the higher risk of all-cause and CVD mortality in postmenopausal females. These findings provide new ideas and targets for the health management of postmenopausal women.

## Introduction

Menopause refers to the cessation of the menstrual cycle due to anovulation, which is an inevitable process of aging [1, 2]. Menopause causes significant fluctuations of sexual hormones in females. The decline of estrogen secreted by the ovaries during menopause may lead to physical discomfort and a series of medical issues, including hot flashes and night sweats, emotional changes, insomnia, urogenital atrophy, osteoporosis, susceptibility to cardiovascular disease and diabetes. The age of menopause varies greatly. The average age of menopause is 51 years old, ranging from 40 to 60 years old [3]. A large amount of studies have indicated that postmenopausal women have a higher risk of cardiovascular disease (CVD) and death [4–6]. Therefore, it is crucial to identify modifiable factors to prevent complications and reduce mortality in postmenopausal women, especially to reduce the risk of cardiovascular death.

Vitamin D is a kind of fat-soluble vitamin that promotes calcium and phosphorus absorption, and 25-hydroxyvitamin D (25(OH)D) is the primary storage in the body [7]. Vitamin D deficiency is highly prevalent among the general populations [8–11]. Serum 25(OH)D deficiency is a common risk factor for various diseases, such as CVD, hypertension, diabetes, cancer, chronic kidney disease, sepsis and so on [12–15]. It is suggested that vitamin D deficiency has been strongly associated with all-cause mortality [16]. In particular, current researches suggested that Vitamin D deficiency is associated with the severity and mortality rate of Coronavirus disease 2019 (COVID-19) cases, which has raised public concern about the association between vitamin D deficiency and health status [17, 18].

Current research suggested that the lack of 25(OH)D increased the risk of fracture across the menopause [19]. It has also been reported that high 25(OH)D concentrations in serum reduced the risk of breast cancer [20], and even low 25(OH)D concentration is associated with lower overall survival rate of patients with ovarian cancer [21]. However, the association between 25(OH)D levels and all-cause and cause-specific mortality in postmenopausal females remains unclear. Based on this, we investigated the relationship between serum 25(OH)D concentrations and all-cause and cause-specific mortality in a nationally representative sample of postmenopausal women in the United States.

## Methods

### Study design and population

National Health and Nutrition Examination Survey (NHANES) is a cross-sectional survey aimed at collecting information on the health and nutritional status of adults and children in the United States, which was conducted by the National Center for Health Statistics (NCHS) of the Centers for Disease Control and Prevention (CDC). All NHANES protocols were approved by the CDC’s National Center for Health Statistics Ethics Review Board, and all participants of survey provided written informed consent.

**Fig. 1:**
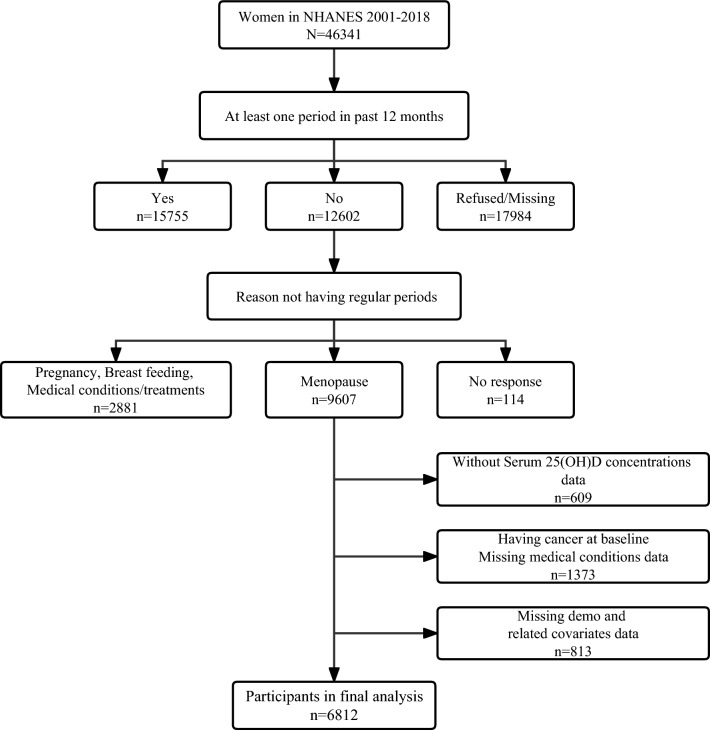
Flow chart of study participants

In this study, seven cycles of NHANES from 2001 to 2018 (2001–2002, 2003–2004, 2005–2006, 2007–2008, 2009–2010, 2011–2012, 2013–2014, 2015–2016, 2017–2018) were selected for further analysis. Menopausal status was determined according to the responses of the questionnaire on reproductive health. Participants were first asked, “Had regular periods in past 12 months?”. The subjects who answered “no” continued to be asked, “Reason not having regular periods. (Options: Menopause/change of life; Pregnancy; Breastfeeding; Medical conditions/treatments; other)”. So first, 9607 postmenopausal women at enrollment were included. After excluding those with missing serum 25(OH)D concentrations (n = 609), having cancer at baseline or missing medical conditions data (n = 1373), missing demo and related covariates data (n = 813), the final sample population for the purposes of this study was 6812 participants (Fig. 1).

### Measurement of serum 25(OH)D concentrations

In the 2001–2006 cycle of the NHANES, serum 25(OH)D concentrations were measured by DiaSorin RIA kit (Stillwater MN) and by a standardized liquid chromatography–tandem mass spectrometry (LC–MS/MS) method in 2007–2018. So as to use and analyze 25(OH)D concentration, the regression equations were selected to converted RIA measurements of 25(OH)D concentration to equivalent 25(OH)D measurements in the standardized LC–MS/MS method to adjust for assay drifts. Follow the recommendations of CDC, LC–MS/MS data was performed for analysis [22].

### Determination of mortality outcomes

To determine the mortality status of the follow-up population, we used 2001–2018 NHANES public-use linked mortality files, the survival status of participants was followed up to December 31, 2019. The ICD-10 was used to determine disease-specific death, and NCHS classified heart diseases (054–068), malignant neoplasms (019–043), and all other causes (010) [23]. Serum 25(OH)D levels were classified according to the Endocrine Society Clinical Practice guidelines [24], as follows: < 25.00 nmol/L, indicating severe vitamin D deficiency; 25.00–49.99 nmol/L, indicating vitamin D deficiency; 50.00–74.99 nmol/L, indicating vitamin D insufficiency; ≥ 75.00 nmol/L, indicating vitamin D sufficiency.

### Covariates

Based on previous research, we used additional covariates in this study. Questionnaires were collected to acquire demographic information (age, race/ethnicity, education level, family income), smoking status, and alcohol intake. Body mass index (BMI, kg/m^2^) were measured at the Mobile Examination Center. The history of hypertension or diabetes obtained from laboratory, examination and questionnaire data.

Race/ethnicity was categorized as Mexican American, other Hispanic, non-Hispanic White, non-Hispanic Black, other Hispanic or other race; education levels were classified as less than a high school education, some high school, high school graduate/GED, some college or associate’s degree, college graduate or more; BMI was categorized as underweight (< 18.5 kg/m^2^), normal (18.5 to < 25 kg/m^2^), overweight (25 to < 30 kg/m^2^), subjects with obesity (BMI 30 kg/m^2^ or greater); alcohol intake was defined by the monthly alcohol consumption, and categorized by (non-drinker, 1 to < 5 drinks/month, 5 to < 10 drinks/month, or 10 + drinks/month; and smoking status was classified as current, former, or never smoker.

Plasma glycohemoglobin (%), glucose (mg/dL), cholesterol (mg/dL), direct HDL-cholesterol (mg/dL), LDL-cholesterol (mg/dL) and triglycerides (mg/dL) were acquired from the NHANES laboratory examination component.

### Statistical analyses

The data in this research were statistically analyzed according to the CDC guidelines [25]. Serum 25(OH)D levels were classified as mentioned above. We presented continuous variables using the mean and standard deviation, and described categorical variables as percentages. And we used three Cox regression models to explore the association of serum 25(OH)D concentrations and mortality: Model 1 (unadjusted); Model 2 was adjusted for age and race/ethnicity; Model 3 was adjusted for age, race/ethnicity, education level, PIR, BMI, smoking status, alcohol intake, hypertension and diabetes.

In addition, restricted cubic spline regression (RCS) model was used to investigate the non-linear relationship between serum 25(OH)D concentration and mortality. And the lowest point of hazard ratios (HRs) in RCS analysis was defined as cut-off value. At last, we conducted subgroup analyses based on age (< 60 years old or ≥ 60 years old), race/ethnicity (Whites or non-Whites), BMI (< 25.00 or ≥ 25.00), hypertension, and diabetes. R version 3.4.3 was used for all statistical analyses.

## Results

### Baseline characteristics of study participants

6812 participants of postmenopausal women were enrolled in this study. The mean age of participants was 61.00 ± 10.86 years old. The weighted mean concentration of serum 25(OH)D was 72.57 ± 29.93 nmol/L; 29.70% of participants had deficient vitamin D (< 50.00 nmol/L), and 65.34% had insufficient vitamin D (< 75.00 nmol/L). The baseline characteristics of the selected female participants according to serum 25(OH)D are presented in the Table 1. Participants who had higher 25(OH)D concentrations were more likely to be older, non-Hispanic White; had higher education levels and family income; were less likely to be subjects with obesity, current smokers and alcohol intake. And higher levels of serum 25(OH)D were also associated with lower incidence rate of hypertension and diabetes.

**Table 1:** Baseline characteristics of participants according to serum 25(OH)D concentrations

Characteristic	Serum 25(OH)D concentrations (nmol/L)					P value
	Total	< 25.00	25.00–49.99	50.00–74.99	≥ 75.00	
Number of participants (%)	6812 (100)	244 (3.58)	1779 (26.12)	2428 (35.64)	2361 (34.66)	
Age (years)	61.00 ± 10.86	59.00 ± 10.42	60.00 ± 11.05	60.00 ± 11.01	62.00 ± 10.57	** < 0.001**
Race/ethnicity (%)						** < 0.001**
Mexican American	1,064 (15.62)	42 (17.21)	381 (21.42)	449 (18.49)	192 (8.13)	
Non-hispanic white	3,252 (47.74)	42 (17.21)	586 (32.94)	1,153 (47.49)	1,471 (62.30)	
Non-hispanic black	1,409 (20.68)	134 (54.92)	571 (32.10)	412 (16.97)	292 (12.37)	
Other hispanic	595 (8.73)	17 (6.97)	144 (8.09)	243 (10.01)	191 (8.09)	
Other race	492 (7.22)	9 (3.69)	97 (5.45)	171 (7.04)	215 (9.11)	
Education (%)						** < 0.001**
Less than 9th grade	999 (14.67)	29 (11.89)	318 (17.88)	417 (17.17)	235 (9.95)	
9-11th grade	1,021 (14.99)	63 (25.82)	325 (18.27)	357 (14.70)	276 (11.69)	
High school graduate/GED	1,738 (25.51)	60 (24.59)	453 (25.46)	591 (24.34)	634 (26.85)	
Some college or AA	1,823 (26.76)	64 (26.23)	455 (25.58)	643 (26.48)	661 (28.00)	
College graduate or above	1,223 (17.95)	28 (11.48)	226 (12.70)	418 (17.22)	551 (23.34)	
Not recorded	8 (0.12)	0 (0.00)	2 (0.11)	2 (0.08)	4 (0.17)	
Family poverty income ratio	3.04 ± 1.61	1.84 ± 1.41	2.49 ± 1.64	2.86 ± 1.60	3.57 ± 1.58	** < 0.001**
BMI (%)						** < 0.001**
Underweight (< 18.5)	88 (1.29)	3 (1.23)	13 (0.73)	28 (1.15)	44 (1.86)	
Normal (18.5 to < 25)	1,701 (24.97)	38 (15.57)	301 (16.92)	568 (23.39)	794 (33.63)	
Overweight (25 to < 30)	2,151 (31.58)	58 (23.77)	500 (28.11)	845 (34.80)	748 (31.68)	
subjects with obesity (30 or greater)	2,872 (42.16)	145 (59.43)	965 (54.24)	987 (40.65)	775 (32.83)	
Smoking status (%)						** < 0.001**
Never smoker	4,095 (60.11)	113 (46.31)	1,027 (57.73)	1,515 (62.40)	1,440 (60.99)	
Former smoker	1,722 (25.28)	60 (24.59)	415 (23.33)	586 (24.14)	661 (28.00)	
Current smoker	991 (14.55)	71 (29.10)	336 (18.89)	326 (13.43)	258 (10.93)	
Not recorded	4 (0.06)	0 (0.00)	1 (0.06)	1 (0.04)	2 (0.08)	
Alcohol intake (%)						** < 0.001**
Non-drinker	2,965 (43.53)	103 (42.21)	865 (48.62)	1,103 (45.43)	894 (36.87)	
1–5 drinks/month	2,505 (36.77)	97 (39.75)	680 (38.22)	903 (37.19)	825 (34.94)	
5–10 drinks/month	226 (3.32)	11 (4.51)	42 (2.36)	84 (3.46)	89 (3.77)	
10 + drinks/month	544 (7.99)	17 (6.97)	96 (5.40)	168 (6.92)	263 (11.14)	
Not recorded	572 (8.40)	16 (6.56)	96 (5.40)	170 (7.00)	290 (12.28)	
Hypertension (%)	3,670 (53.88)	150 (61.48)	986 (55.42)	1,241 (51.12)	1,293 (54.76)	0.051
Diabetes (%)	1,626 (23.87)	88 (36.07)	516 (29.01)	558 (22.98)	464 (19.65)	** < 0.001**

Data are presented as mean ± SD or n (%)

In addition, we also explored the relationship between cardiometabolic biomarkers and serum 25(OH)D. As shown in Table 2, the levels of serum 25(OH)D were negatively associated with the levels of glycohemoglobin, glucose and triglycerides, and positively associated with HDL levels at baseline.

**Table 2:** Baseline levels of cardiometabolic markers according to serum 25(OH)D concentrations among participants

	Serum 25(OH)D concentrations (nmol/L)				P value
	< 25.00	25.00–49.99	50.00–74.99	≥ 75.00	
Glycohemoglobin (n = 6794) (%)	5.80 ± 1.19	5.70 ± 1.25	5.60 ± 0.93	5.50 ± 0.76	** < 0.001**
Glucose (n = 3354) (mg/dL)	106.00 ± 49.72	102.00 ± 41.92	101.00 ± 28.62	100.00 ± 24.21	** < 0.001**
Cholesterol (n = 6759) (mg/dL)	196.00 ± 39.21	206.00 ± 42.95	211.00 ± 40.09	208.00 ± 40.99	** < 0.001**
LDL (n = 3256) (mg/dL)	111.00 ± 30.64	119.00 ± 38.07	123.00 ± 35.93	118.00 ± 37.16	**0.002**
HDL (n = 6758) ((mg/dL))	53.00 ± 18.31	53.00 ± 16.06	57.00 ± 16.64	61.00 ± 18.43	** < 0.001**
Triglycerides (n = 3319) (mg/dL)	107.42 ± 77.10	119.00 ± 83.19	121.00 ± 100.14	106.00 ± 85.09	**0.001**

Mean ± SD for continuous variables: P-value was calculated by Wilcoxon rank-sum test for complex survey samples

### Association of 25(OH)D concentration with mortality

During the follow-up of this study, 1448 all-cause deaths occurred, including 393 CVD-related deaths and 263 cancer deaths (Table 3). We constructed three Cox regression models to explore the independent effect of serum 25(OH)D levels in mortality. The multivariate adjustments including age, race/ethnicity, education level, PIR, BMI, smoking status, alcohol intake, hypertension and diabetes. As shown, multivariate adjusted hazard ratios (HRs) and 95% confidence intervals (CIs) from lowest to highest serum 25(OH)D categories (< 25.00, 25.00–49.99, 50.00–74.99, and ≥ 75.00 nmol/L) were 1.00 (reference), 0.63 (0.44, 0.92), 0.50 (0.34,0.71), and 0.46 (0.31,0.69), respectively, for all-cause mortality (Model 3). While for CVD mortality, the multivariate adjusted HRs and 95% CIs were 1.00 (reference), 0.34 (0.19, 0.61), 0.40 (0.22, 0.74), and 0.60 (0.31,1.15), respectively. In addition, we also explored the relationship between serum 25(OH)D concentrations and cancer mortality. The results showed the HRs and 95% CIs were 1.00 (reference), 0.87 (0.45, 1.70), 0.72 (0.38, 1.36), and 1.36 (0.67, 2.73). Compared with the group of serum 25(OH)D < 25.00 nmol/L, postmenopausal females with higher levels of serum 25(OH)D (≥ 25.00 nmol/L) had lower all-cause and CVD mortality. Although there are statistical differences in trend for cancer mortality, there is no statistical difference among groups, which may be due to the small sample size.

**Table 3:** HRs (95% CIs) for mortality according to serum 25(OH)D concentrations among participants

	Serum 25(OH)D concentrations (nmol/L)				P value
	< 25.00	25.00–49.99	50.00–74.99	≥ 75.00	
All-cause mortality					
Number of deaths (%)	72 (29.51)	448 (25.18)	521 (21.46)	407 (17.24)	
Model 1 HR (95% CI) P-value	1.00	0.70 (0.51,0.95) **0.025**	0.55 (0.41,0.74) < **0.001**	0.52 (0.37,0.73) < **0.001**	** < 0.001**
Model 2 HR (95% CI) P-value	1.00	0.58 (0.41,0.83) **0.003**	0.41 (0.29,0.58) < **0.001**	0.37 (0.25,0.54) < **0.001**	** < 0.001**
Model 3 HR (95% CI) P-value	1.00	0.63 (0.44,0.92) **0.017**	0.50 (0.34,0.71) < **0.001**	0.46 (0.31,0.69) < **0.001**	** < 0.001**
CVD mortality					
Number of deaths (%)	23 (9.43)	115 (6.46)	146 (6.01)	109 (4.62)	**0.003**
Model 1 HR (95% CI) P-value	1.00	0.34 (0.20,0.58) < **0.001**	0.41 (0.24,0.71) **0.001**	0.63 (0.35,1.13) 0.12	**0.020**
Model 2 HR (95% CI) P-value	1.00	0.35 (0.19,0.63) < **0.001**	0.38 (0.21,0.68)** 0.001**	0.59 (0.30,1.13) 0.11	**0.029**
Model 3 HR (95% CI) P-value	1.00	0.34 (0.19, 0.61) < **0.001**	0.40 (0.22,0.74) **0.003**	0.60 (0.31,1.15) 0.12	
Cancer mortality					
Number of deaths (%)	19 (7.79)	81 (4.55)	86 (3.54)	77 (3.26)	
Model 1 HR (95% CI) P-value	1.00	0.97 (0.52,1.81) 0.92	0.85 (0.46,1.58) 0.61	1.36 (0.70,2.64) 0.40	**0.015**
M odel 2 HR (95% CI) P-value	1.00	0.90 (0.44,1.88) 0.79	0.74 (0.36,1.52) 0.42	1.17 (0.55,2.49) 0.69	0.070
Model 3 HR (95% CI) P-value	1.00	0.87 (0.45,1.70) 0.69	0.72 (0.38,1.36) 0.31	1.36 (0.67,2.73) 0.40	**0.004**

Model 1: Non-adjusted

Model 2: Adjusted for age, race/ethnicity

Model 3: Adjusted for age, race/ethnicity, education level, PIR, BMI, smoking status, alcohol intake, hypertension and diabetes

### Results of nonlinear of 25(OH)D concentration and mortality

**Fig. 2:**
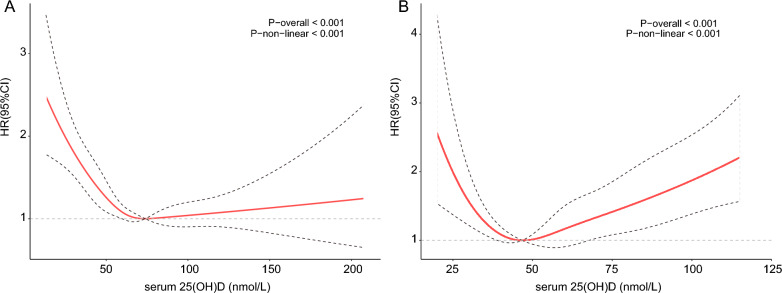
Association between 25(OH)D concentration and all-cause (**A**) and CVD mortality (**B**) in postmenopausal women. Adjusted for age, race/ethnicity, education level, PIR, BMI, smoking status, alcohol intake, hypertension and diabetes. The solid and dotted lines represent the estimated values and their corresponding 95% CIs, respectively

By using the restricted cubic spline regression (RCS) models with full adjustment for confounders, we found that there was the L-shaped association between serum 25(OH)D concentrations and all-cause mortality, while serum 25(OH)D levels displayed a U-shaped relationship with CVD mortality (Fig. 2). And the cut-off value for all-cause mortality were 73.89 nmol/L, and 46.75 nmol/L for CVD mortality.

### Stratified analyses

**Fig. 3:**
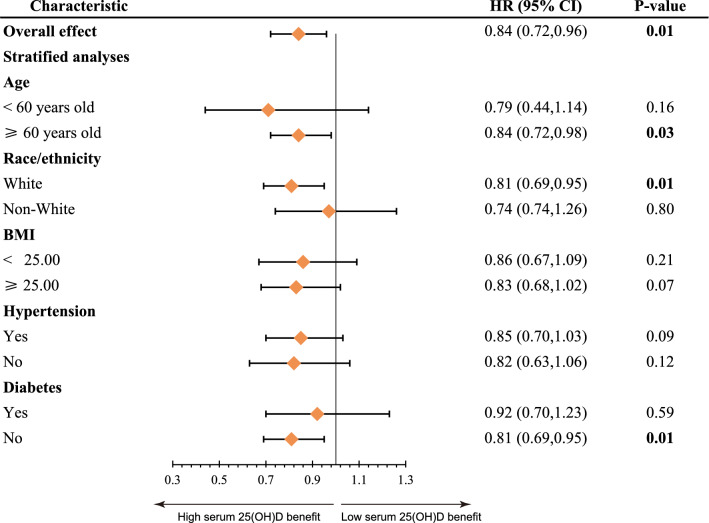
Forest plots of stratified analyses of serum 25(OH)D and all-cause mortality. Adjusted for age, race/ethnicity, education level, PIR, BMI, smoking status, alcohol intake, hypertension and diabetes, except the variable itself

The data of Fig. 3 showed the association of serum 25(OH)D concentrations and all-cause mortality as stratified by age, race, BMI, and race/ethnicity, history of hypertension, and history of diabetes. In subgroup analysis, lower serum 25(OH)D levels (< 73.89 nmol/L) and higher 25(OH)D concentrations (≥ 73.89 nmol/L) present similar advantages for survival rate among postmenopausal females. Additionally, our results showed a stronger inverse relationship between serum 25(OH)D concentrations and all-cause mortality in older, white, with no history of diabetes postmenopausal women.

## Discussion

In this large prospective cohort study, we revealed the association between serum 25(OH)D concentrations and all-cause and CVD mortality of postmenopausal women. The results indicated a L-shaped relationship between serum 25 (OH) D levels and all-cause mortality for postmenopausal females, while it seems to be a U-shaped with CVD mortality. That means within a certain range, lower serum 25 (OH) D levels were significantly associated with higher risk of all-cause and CVD mortality.

According to Clinical Practice guidelines of Endocrine Society, our result indicated that 65.34% of postmenopausal females have suffering serum 25(OH)D insufficiency, which means vitamin D deficiency was commonly present in women of postmenopausal. And above result is consistent with previous researches [26–28]. Our data also suggested that compared to postmenopausal women with serum 25(OH)D ≥ 25.00 nmol/L, those with serum 25(OH)D < 25.00 nmol/L had higher all-cause mortality and CVD mortality.

Vitamin D deficiency may have adverse effects on the immune system and increase the risk of acute respiratory infections. The mortality rate of COVID-19 patients in recent years is positively correlated with Vitamin D deficiency [17, 18]. And another meta-analysis indicated that COVID-19 positive patients have lower serum Vitamin D concentrations, which is more prominent in women [29]. Although there is currently no research to elucidate the relationship between vitamin D concentration and the onset and progression of COVID-19 in postmenopausal women, it is still recommended that supplementing vitamin D may reduce the severity of COVID-19 infection [30].

Currently, a series of studies suggested that low levels of 25(OH)D in serum were closely related to a higher risk of mortality, but the optimal concentration of serum 25(OH)D remained controversial. The American Institute of medicine suggested that 50.00 nmol/L was sufficient for bone health [31]. However, the Endocrinology Society proposed that the optimal concentration of 25(OH)D in serum among general adults should be at least 75.00 nmol/L for better health condition [28]. A study put forward that the thresholds of serum 25(OH)D was 27.70 and 54.40 nmol/L for CVD and all-cause mortality respectively in American patients with osteoarthritis [28]. But another meta-analysis suggested that with the increase of circulating 25(OH)D, the mortality risk showed a non-linear decrease, and the optimal concentration was about 75.00–87.50 nmol/L [32]. The reasons for the above controversy may be due to differences in the target population, sample size, and basic health status. Lack of vitamin D may worsen menopausal symptoms, but the evidence is not sufficient [30]. And insufficient Vitamin D can affect the bone health and exacerbate osteoporosis in postmenopausal females [30, 33]. But no research has yet focused on the relationship between 25(OH)D and mortality rate among postmenopausal women. The data of this study indicated that lower 25(OH)D levels in serum of postmenopausal women may lead to a higher risk of all-cause and CVD mortality. And the values corresponding to the lowest all-cause and CVD mortality rate are 73.89 nmol/L and 46.75 nmol/L, respectively.

The relationship between vitamin D and cancer mortality is uncertain [34–37]. Cervical, ovarian, and uterine cancer are the three most common types of gynecologic cancers. The relationship between vitamin D exposure and survival rate of ovarian cancer survivors is controversial [24, 38, 39]. And serum 25(OH)D does not seem to improve the prognosis of uterine cancer [40, 41]. Although a meta-analysis showed that circulating 25(OH)D was associated with overall mortality in in stage I-IIIa postmenopausal breast cancer patients [42], there was no significant correlation between serum 25(OH)D levels and cancer mortality in postmenopausal women in our study. The reason for the above results may be due to the insufficient sample size for cancer in this study, a larger scale prospective study is needed to investigate the relationship between vitamin D and cancer mortality.

To further identify the population at higher risk of all-cause mortality in postmenopausal females, we conducted a sub layer analysis. The results showed that higher serum vitamin D concentrations (≥ 73.89) had a better advantage on all-cause mortality in the elderly (≥ 60 years old), Whites, and without a history of diabetes populations. The absorption and utilization of Vitamin D vary among different races [43]. Previous study proposed that non-Hispanic Blacks with a higher proportion of lower serum 25(OH)D below 25.00 nmol/L in the osteoarthritis patients [28], which is consistent with our results of postmenopausal women. However, White people seem to have poorer adaptability to vitamin D deficiency. An analysis of the American population suggested that 25-hydroxyvitamin D deficiency was related to an increased risk of fatal stroke in Whites but not Blacks [44]. And lower serum 25(OH)D concentrations appeared to hurt more in Whites osteoarthritis patients [28]. Consistent with the above results, our study also showed that the lack of vitamin D in Whites postmenopausal women is more correlated with a higher risk of all-cause mortality. It is worth noting that elderly postmenopausal females with low levels of serum 25(OH)D have a higher risk of death. The mechanism of the above results is still unclear, but in the process of clinical health management, more attention should be paid to the elderly and Whites population.

The potential mechanism for the association between lower 25(OH)D levels and increased risk of death is currently unclear. From a biological perspective, a lot of tissues and cells respond to 25(OH)D [45, 46]. 25(OH)D is a kind of self-balancing regulator of the renin angiotensin aldosterone system, which can affect blood pressure [47]. And Vitamin D can influence the migration and differentiation of macrophages and the uptake of cholesterol, inhibit the formation of foam cells, and reverse the cholesterol metabolism that causes atherosclerosis among diabetes patients, which may increase the risk of cardiovascular disease in related populations [48, 49]. Vitamin D deficiency is related with an increased risk of cardiovascular events (including metabolic syndrome, type 2 diabetes mellitus and dyslipidemia) [30], thus, we also explored the relationship between the index of cardiovascular metabolism and serum 25(OH)D in postmenopausal women. Higher serum 25(OH)D levels were significantly associated with lower levels of glycohemoglobin and glucose, and with high level of HDL. A large number of studies indicated that low concentrations of vitamin D were related with CVD, including coronary calcification and elevated triglyceride levels [50, 51]. Our study suggests that lower levels of serum 25(OH)D levels are associated with CVD mortality in postmenopausal women. Interestingly, our results indicated that the optimal protective concentration of 25(OH)D for CVD related death in postmenopausal females was 46.75 nmol/L. That is to say, when the serum 25(OH)D concentration is higher than 46.75 nmol/L, the risk of CVD death increases. There is currently controversy over whether vitamin D is a protective factor for cardiovascular health. As mentioned above, a large number of studies have proposed that high 25(OH)D can reduce the risk of CVD and CVD related mortality, but there are also studies that suggest that excessive supplementation of vitamin D may increase the risk of cardiovascular events [52–54]. The potential biological explanation may be that high concentration of 25(OH)D leads to high blood calcium concentration, which eventually leads to vascular calcification, atherosclerosis and hypercoagulability [55, 56]. Thus, determining the most suitable serum 25(OH)D concentration is crucial. Unfortunately, this article is currently unable to define the relationship between serum 25(OH)D concentration and blood calcium, which will be the focus of our next research.

In addition to cardiovascular factors, osteoporosis is another major cause of death in postmenopausal females. Postmenopausal women experience rapid bone loss due to decreased levels of sex hormones caused by ovarian disfunction, which affects calcium metabolism and increases the possibility of osteoporosis. The probability of osteoporosis in postmenopausal women significantly increases, and it seriously endangers health and lifespan [57]. The decrease in estrogen levels after menopause can cause cardiovascular symptoms and osteoporosis. Estrogen deficiency can induce the loss of cancellous and cortical bone in menopause [58]. The use of hormone replacement therapy in menopause can reduce the risk of osteoporosis, but it is related to the increased risk of cardiovascular and cerebrovascular events, breast cancer and other adverse health outcomes [59, 60]. Previous studies had indicated that Vitamin D supplementation can prevent osteoporosis and fractures in postmenopausal women [61–64]. Due to current article lacks complete information on osteoporosis and serum estrogen, whether Vitamin D reduces postmenopausal mortality is due to the reduction of osteoporosis, and whether this process is mediated by estrogen, requires further research in the future.

## Conclusions

In conclusion, this study is the first to explore the relationship between serum 25 (OH) D and all-cause mortality and specific mortality in postmenopausal women. Lower serum 25(OH)D concentrations were significantly and nonlinearly associated with a higher risk of all-cause and CVD death among postmenopausal females in the United States. These data provide new clues for the health management of postmenopausal females. For postmenopausal women, regular testing of blood serum 25(OH)D concentrations may be necessary, and based on the results, it is recommended to sunlight exposure supplement, or even Vitamin D supplementation.

## Data Availability

Data described in the manuscript are publicly and freely available without restriction at https://www.cdc.gov/nchs/nhanes/index.htm.

## Abbreviations

25(OH) D: 25-Hydroxyvitamin D
NDI: National Death Index
CVD: Cardiovascular disease,
LC–MS/MS: Liquid chromatography–tandem mass spectrometry
BMI: Body mass index
